# An Interactive Pain Application (MServ) Improves Postoperative Pain Management

**DOI:** 10.1155/2021/8898170

**Published:** 2021-04-02

**Authors:** Richard Gordon-Williams, Andreia Trigo, Paul Bassett, Amanda Williams, Stephen Cone, Martin Lees, Brigitta Brandner

**Affiliations:** ^1^University College London Hospitals NHS Foundation Trust, Department of Anaesthesia, London NW1 2BU, UK; ^2^Stats Consultancy Ltd., 40 Longwood Lane, Amersham, Bucks HP7 9EN, UK; ^3^University College London, Clinical, Educational & Health Psychology, Gower St, London WC1E 6BT, UK; ^4^Barts Health NHS Trust, Department of Cardiothoracic Anaesthesia, London EC1A 7BE, UK

## Abstract

**Background:**

Most patients have moderate or severe pain after surgery. Opioids are the cornerstone of treating severe pain after surgery but cause problems when continued long after discharge. We investigated the efficacy of multifunction pain management software (MServ) in improving postoperative pain control and reducing opioid prescription at discharge.

**Methods:**

We recruited 234 patients to a prospective cohort study into sequential groups in a nonrandomised manner, one day after major thoracic or urological surgery. Group 1 received standard care (SC, *n* = 102), group 2 were given a multifunctional device that fed back to the nursing staff alone (DN, *n* = 66), and group 3 were given the same device that fed back to both the nursing staff and the acute pain team (DNPT, *n* = 66). Patient-reported pain scores at 24 and 48 hours and patient-reported time in severe pain, medications, and satisfaction were recorded on trial discharge. *Findings*. Odds of having poor pain control (>1 on 0–4 pain scale) were calculated between standard care (SC) and device groups (DN and DNPT). Patients with a device were significantly less likely to have poor pain control at 24 hours (OR 0.45, 95% CI 0.25, 0.81) and to report time in severe pain at 48 hours (OR 0.62, 95% CI 0.47–0.80). Patients with a device were three times less likely to be prescribed strong opioids on discharge (OR 0.35, 95% CI 0.13 to 0.95). *Interpretation*. Using an mHealth device designed for pain management, rather than standard care, reduced the incidence of poor pain control in the postoperative period and reduced opioid prescription on discharge from hospital.

## 1. Introduction

Good pain control is integral to rehabilitation after surgery, [1] but poor pain control continues to be reported by up to 70% of patients, despite improvements in both clinical and organisational factors in the management of perioperative pain [2]. Severe pain in the postoperative period is a risk factor for poorer postoperative outcome and quality of life, including increased healthcare use and delayed discharge and return to work, as well as the development of chronic postsurgical pain [2]. Strong opioids continue to be the cornerstone of good postoperative pain management, but overuse and underuse of analgesics are both common [2–4]. Overprescription of perioperative opioids in hospital, in a protocolised manner not tailored to the patient's needs, [3, 4] has been identified as a common precursor of chronic opioid use [5, 6]. Acute postoperative pain services play a key role in balancing the risks of poorly controlled postsurgical pain with those of chronic opioid use.

Poor postoperative pain experience is often attributable to pain not being adequately acknowledged or assessed, delayed pain relief, or poor control with or without analgesia [7–9]. These all contribute to patient distress, poor sleep, slower recovery, and poor relationships with the healthcare team [10–12]. Psychological factors are key in pain experience and in pain processing, [13–15] particularly attention to pain being displaced by other concerns and interests, and opportunities for cognitively engaging and emotionally satisfying activities, both of which shift descending modulation of pain away from excitation and towards inhibition, [16] achieving improved pain management by nonpharmacological means [17]. Devices such as patient controlled analgesia (PCA) have improved satisfaction [18, 19] due to the immediacy of pain relief and responsiveness to need. Electronic health devices may offer more frequent pain reporting than reliance on ward nurses' routine observations [20] and can encourage pleasurable engagement and information use [21], ideally reducing pain, distress, and medication requirements. Additionally, direct feedback of poor pain control to clinicians in the postoperative patient represents an opportunity to optimise analgesia, improve pain control, and reduce time in severe pain.

The term mobile health (mHealth) has been defined by the WHO as “medical and public health practice supported by mobile devices” and covers applications (apps) that provide information, symptom monitoring, and advice [22].

mHealth represents a burgeoning field for pain management, allowing patients to access pain advice and interact with their healthcare teams. Importantly, few apps have either engaged healthcare expertise in their development, been rigorously tested to assess pain outcomes, or been shown to be beneficial in the management of pain [23–25].

Two recent systematic reviews assessing the efficacy of apps in pain outcomes found that studies were too heterogeneous to draw appropriate combined conclusions [23, 26]. Thurnheer and colleagues did conclude however that apps may improve pain intensity and quality of life [26]. Furthermore, neither of these systematic reviews found studies on patients with acute postoperative pain and therefore their findings lack generalisability to the perioperative setting.

Since these reviews, Toelle et al. have found that an app-based multidisciplinary programme for the treatment of back pain was superior in reducing pain intensity but not function at 12 weeks when compared to online education and physiotherapy [27]. The authors hypothesised that this may be due to the tailored nature of the content and the increased user engagement after finishing physiotherapy. Bespoke treatment on the basis of user feedback represents an exciting opportunity to improve patient care. Suso-Ribera and colleagues showed that alerting clinicians to adverse symptoms entered remotely by patients with chronic pain led to a clinically significant improvement in a range of outcomes including depression, anxiety, pain interference, and severity when compared to usual treatment [28].

For postoperative pain, reducing distress and increasing patient confidence in treatment constitute nonpharmacological means of improving control. An app-based pain management programme for children with juvenile arthritis reduced pain intensity, but no control arm was used [29]. A postoperative version of this app and another app for the management of paediatric postoperative pain were well accepted but have yet to be assessed for effect on outcomes [30–32]. A proof-of-concept study in adults by Thiel et al. showed that patients' recording of postoperative pain was easy via an app, reporting similar pain severity to that recorded by nursing staff [33]. We have previously reported that patients reported less anxiety when using a previous version of the app after surgery [20]. This study introduces a multifunctional application (MServ) that allows patients access to information tailored to their surgical pathway. On opening the app, the patient logged his or her pain score at rest and on movement: this provided access to further content: “My Operation” (media providing information about the surgery and postoperative journey, e.g., management of chest drains); “Self Help” (including mindfulness and guided breathing exercises); “Guided Physiotherapy” (relevant to the operation type); “Pain Information” (including education about the psychological and biological mechanisms of pain and the tools used in its management, e.g., using PCA); and “Entertainment” (audiobooks and puzzles).

Patient education about the perioperative journey and pain experience via MServ may help to improve pain by reducing anxiety, as well as providing distraction. Furthermore, the opportunity to feed back pain scores to healthcare professionals may improve the sense of involvement with pain treatment, improving satisfaction. Lastly, feedback of pain scores to healthcare professionals enables them to improve responsiveness to pain, tailoring appropriate escalation and deescalation of analgesia in a timely manner.

The primary objective was to assess the efficacy of the device in reducing the incidence of moderate or severe pain (greater than 1 on a 0–4 scale) after 24 and 48 hours compared to standard care. Secondary objectives were as follows: (2A) to assess the effect of the device on the proportion of time in severe pain and patient satisfaction with pain treatment in 48 hours postoperatively, compared to standard care; (2B) to compare the effect of device on prescription of opioid medication on discharge and length of stay with standard care; (2C) to assess the patient experience of and satisfaction with the device; and (2D) to compare pain and satisfaction between patients using a device to feed back pain scores to nursing team alone or to nursing team and pain team, with the latter promoting access to more specialist advice on pain management.

## 2. Methods

### 2.1. Participants

Patients were recruited between March 2017 and May 2018 at a single site specialist urological and thoracic surgical unit at University College London Hospital (UCLH) National Health Service Trust, a tertiary care hospital in London, UK. The study inclusion criteria were as follows: inpatient at trial centre; age 18–80; English speaking; and capacity to use the device. Exclusion criterion was expected inpatient stay less than 48 hours. Patients undergoing all types of thoracic and urological surgery that met these criteria were eligible for study.

Local audit revealed that 50% of patients had a pain score of greater than 1, where 0 represented no pain and 4 represented very severe pain. The incidence of moderate (score of 2) or severe or very severe pain (score of 3 or 4, respectively) in local audit is not unusual for the UK [34]. Studies of perioperative analgesic interventions show small improvements in pain, with PCA showing up to a 17% improvement in VAS in the thoracic population [18]. In order to detect a 20% difference between groups with 80% power and 5% significance, 93 patients were needed in the control group and 66 patients in each of the two device groups.

### 2.2. Intervention

The study received approval from the local Research & Ethics Committee (IRAS reference: 184823). Patients were given information by nurses during routine assessment one to two weeks before surgery. In a previous pilot study [20], it was found that nursing staff behaviour was altered in the presence of a device. Since staff could not be blinded to the use of the device, and the three comparison groups could not be isolated from one another, patients were recruited in series into three consecutive groups (Group 1, March–August 2017; Group 2, August–December 2017; Group 3, January–May 2018) by the research nurse 24 hours after surgery. Patients could withdraw from the trial at any time.

**Figure 1:**
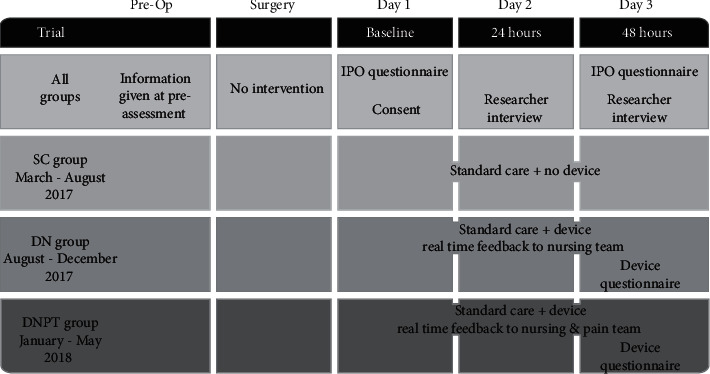
Trial timeline. Patients were recruited to three groups at postoperative day 1 (baseline) and followed for 48 hours (postoperative days 2 and 3). SC: standard care; DN: device with feedback to nursing staff; DNPT: device with feedback to nursing staff and pain team; IPO: International Pain Outcome Questionnaire.

The trial followed patients from postoperative days 1 to 3 (Figure 1). Group 1 received standard care (SC); group 2 received standard care and the device, which provided their pain score to ward nursing staff (device: nursing staff, DN); group 3 received standard care and the same device, which provided their pain score to ward nursing staff *and* to the specialised pain team (device: nursing staff and pain team, DNPT). However, device-recorded pain was only passed on to ward staff and pain team between 8 am and 5 pm, Monday–Friday, as it was relayed by a nurse working those hours. The software and content on the device for both the DN and DNPT groups were identical, and therefore, the experience of using it did not differ between DN and DNPT groups. All patients received standard care, which included reporting of pain scores at rest and on movement on enquiry by nursing staff every 4 hours, as per hospital policy. Furthermore, patients in all groups with PCA or regional anaesthetic infusion were reviewed routinely by the pain team each morning making recommendations for the titration of PCA or regional analgesia. Additionally, the pain team would review patients' analgesic requirements at the request of the medical or nursing team.

### 2.3. Measures

At our institution, patients report pain and nurses record pain as follows: none = 0; mild = 1; moderate = 2; severe = 3; very severe = 4. For consistency, this scale was also used to self-report pain on the device and was used by the research team in patient interviews for assessment of our primary outcome: worst pain in previous 24 hours. Device-reported pain scores were not used in the analysis as patients provided different numbers of scores at different timepoints, complicating any comparison. Additionally, patients have been shown to self-report higher scores, and to do so more frequently, on an electronic device than to nurses [20]. Therefore, all outcome data were derived from patient interview by the research team, not from data recorded by healthcare staff or submitted on the device.

Baseline characteristics of age, sex, surgery type, surgery severity (as defined by AXA-PPP procedure categories), preoperative prescribed medications, baseline pain score (0–4), postoperative baseline analgesia (as defined by a modified analgesic ladder, Table 1), and International Pain Outcome Questionnaire (IPOQ) were collected at trial entry. Admission medication history was verified by the hospital pharmacist as per standard care. Frequency of medications prescribed was recorded according to the following groups: simple analgesics; weak opioids; strong opioids (see Supplementary Information Table 1 for definitions of strong and weak opioids); medications recommended for first-line treatment of neuropathic pain (pregabalin; gabapentin; amitriptyline; duloxetine); [35] other antidepressants; other antiepileptics; benzodiazepines and Z-drugs; and antipsychotics.

Outcomes used in this study can be found in Table 2. Primary outcome was the occurrence of poor pain control, defined as worst pain in the preceding 24 hours of greater than one (on the 0–4 scale) as reported to the research team at 24 and 48 hours after recruitment.

At hospital discharge or at 48 hours after recruitment, whichever was earlier, pain outcomes were reported to the research team using the International Pain Outcome Questionnaire (IPOQ) [36]. The IPOQ consists of 20 questions assessing key variables of postoperative pain, pain intensity, physical and emotional functional interference, side effects, and perceptions of care, with good discriminatory validity [36]. Secondary outcomes were patient-reported percentage of time spent in severe pain and patient satisfaction with pain treatment, as reported to the research team in the exit IPOQ.

### 2.4. Device Characteristics

Patients in device groups (DN and DNPT) were given a smart tablet (Samsung, Android Operating System) to self-report pain scores for 48 hours after recruitment, as often as they wished. The device contained bespoke software (MServ) in kiosk mode as the sole application. MServ was developed by the multidisciplinary pain and surgical team at UCLH in collaboration with Mvine Ltd, London, UK. Patients were familiarised with the device prior to use. The device operated via a wireless encrypted connection to a secure server, and all data was stored encrypted. The Data Protection Act 1998 (UK) and National Health Service code of confidentiality were observed.

Patients were prompted to submit a pain score at rest and on movement when starting the device but were able to do so at any time via an icon always present on the screen. Entering a pain score provided access to a variety of content tailored to the patient's surgical speciality and pain management (Table 3). Only pain scores greater than one out of four were fed back to nursing staff and pain team, according to allocated group. Due to restrictions in integration with existing infrastructure, notification of poor pain control to healthcare teams occurred via an electronic message to the research nurse who immediately informed the appropriate team. For this reason, pain scores submitted outside 0800–1700 weekdays were fed back the next working day.

### 2.5. Statistical Methods

The analyses compared the main study outcomes between the SC group and the pooled device group or DN and DNPT at each timepoint. Binary outcomes were analysed using Fisher's exact test, whilst group comparisons were made using parametric or nonparametric tests, according to normality of distribution.

To adjust for possible confounding in data, outcomes were analysed using regression, controlling for characteristics of the three groups where there were differences. Binary outcomes were analysed using logistic regression, whilst continuous outcomes were analysed using multiple linear regression. Histograms of raw data were plotted to visually assess for skewedness (SPSS skewness score > 1.5). Due to the positively skewed distribution of the continuous outcomes, these were log-transformed to allow parametric analysis.

## 3. Results

### 3.1. Descriptive Data

**Figure 2:**
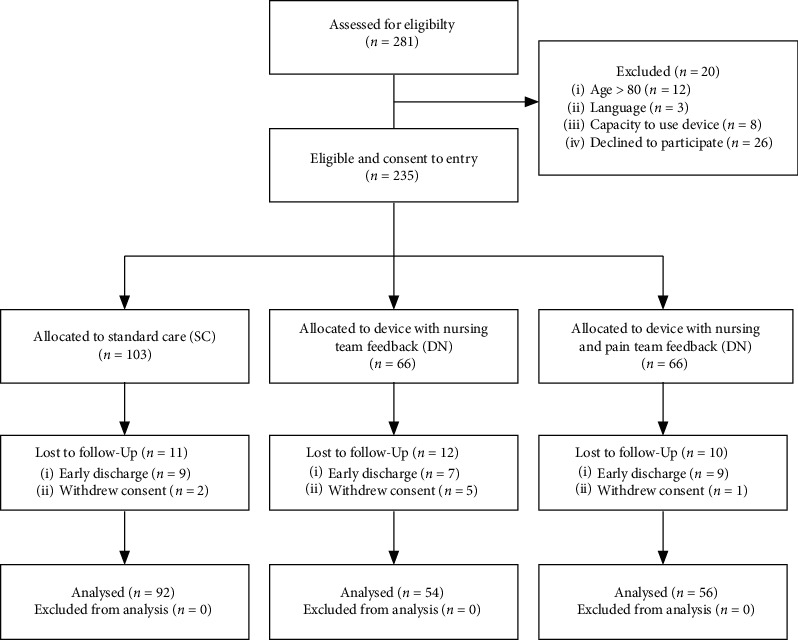
CONSORT flow diagram of trial recruitment and follow-up.

234 patients were recruited, of whom 11 (11%) assigned to SC, 12 (18%) to DN, and 10 (15%) to DNPT did not complete the trial (Figure 2). Characteristics such as age, sex, surgical complexity, and preadmission opioid use were comparable across all groups (Table 4). Device groups had higher proportions of thoracic surgical type (SC group: 75%; device groups: 94%), less percentage time reported in severe pain at baseline (SC: median 30% [IQR 10–70], device: 10% [0–30]), and more patients on step 4 analgesia (SC: 75%; device: 96%). These differences were adjusted for in later analyses. The frequency of prescription of simple analgesics, antineuropathic agents, antidepressants, benzodiazepines, and Z-drugs did not differ between groups prior to admission (see Supplementary Information Table 2 for medication use prior to hospital admission).

### 3.2. Primary Outcome: Device Effect on Incidence of Moderate or Severe Pain after 24 and 48 Hours

Pain control improved over time across both standard care and device groups from trial entry on the first postoperative day to trial exit on the third postoperative day or discharge (Table 5). In the first 24 hours, those in the device groups were half as likely to have uncontrolled pain as those in standard care group (OR 0.45 95% CI 0.25, 0.81) (Table 5). No difference was found between device groups and standard care group at 48 hours, with a much larger range of possible values.

### 3.3. Secondary Outcome: Device Effect on Proportion of Time in Severe Pain and Patient Satisfaction with Pain Treatment in 48 Hours Postoperatively

Patient-reported time spent in severe pain, expressed as a percentage, was greater in SC (median 10%, IQR 10–40%), compared to those with a device (0%, IQR 0–10%), at 48 hours. Ratio of patient-reported time spent in severe pain for all those with a device compared to SC after adjusting for pain at baseline, age, speciality, and PCA use was 0.62 (95% CI 0.47–0.80, *p* < 0.001). Unadjusted data also indicated improved patient-reported time spent in severe pain in those with a device when compared to standard care group.

Satisfaction with pain treatment was high in all groups. On a scale of 0–10, satisfaction was rated as mean of 8.3 (SD 2.5) in SC and 8.9 (SD 1.6) in those with a device. When adjusted for baseline satisfaction, speciality, age, and PCA use, this represents a mean difference of 0.8 (95% CI 0.2, 1.3, *p* = 0.006) in favour of those with a device. Other measures derived from the International Pain Outcome Questionnaire administered after 48 hours with a device are reported in Table 6.

### 3.4. Secondary Outcome: Device Effect on Opioid Medication on Discharge and Length of Stay

Frequency of opioid prescription on discharge in each group and odds of opioid prescription adjusted for baseline opioid prescription are shown in Table 7. Prescription of any opioid was more common in the device groups than in standard care, but that of strong opioid was more common in standard care than in combined device groups. Frequency of prescription of nonopioid analgesics, other antidepressants, other antiepileptics, benzodiazepines and Z-drugs, and antipsychotics did not differ at discharge (see Supplementary Information Table 3 for medication prescription at hospital discharge). Length of stay was equivalent in all the three groups (SC, 6.7 ± 5.4 days; both device groups, 6.5 ± 5.59; DN, 6.7 ± 6.1; DNPT, 6.3 ± 5.0; *p* = 0.94).

### 3.5. Secondary Outcome: Patient Experience and Satisfaction with Device

In both device groups, two-thirds of patients stated they were pleased, and none was displeased, with the device. Overall, 61% of patients found the device easy or very easy to use, with less than 10% finding it difficult or very difficult. Satisfaction with the device was rated a mean of 7.0/10 (SD 2.1); 60% of patients reported using the nonpharmacological content on the device to help with pain. Ratings of device content (0–10) were as follows: surgery information 9.0 ± 1.5; pain information 8.8 ± 1.8; guided stretch 8.7 ± 2.3; guided relaxation 8.3 ± 2.3; and games and puzzles 8.2 ± 2.9.

### 3.6. Secondary Outcome: Feedback of Poorly Controlled Pain to Healthcare Professionals on Pain Control, Satisfaction at 24 and 48 Hours, and Opioid Use at Discharge

Pain control improved from 24 hours to 48 hours in all groups. Logistic regression analysis was conducted taking the proportion of patients with a pain score >1 as the dependent variable and group (SC, DN, and DNPT) as the independent variable, controlling for age, baseline pain, PCA use, and surgical speciality, at 2 timepoints: 24 hours and 48 hours. Pain control at 24 hours did not differ for DN compared with SC but showed a clear advantage for the DNPT group over SC, with the odds of poor pain control being three times lower. Pain control at 48 hours did not differ between either device group and SC (Table 8).

Further analysis showed similar patient-reported time spent in severe pain for DN (median 0%, IQR 0–10%) and DNPT groups (0%, 0–10%). Ratio of patient-reported time spent in severe pain in DN and DNPT groups compared to SC after adjusting for pain at baseline, age, speciality, and PCA use was 0.61 (95% CI 0.44–0.83, *p* = 0.002) in the DN group and 0.54 (0.39–0.75, *p* > 0.001) in the DNPT group, representing a 39% and 46% reduction in time patients reported in severe pain, respectively.

Satisfaction scores were mean 8.6 (SD 1.8) in DN and 8.5 (SD 1.7) in DNPT groups. Once adjusted for baseline satisfaction, age, and speciality, mean satisfaction at end of trial was +0.7 (0.0, 1.4) in DN (*p* = 0.04) and +0.8 (0.1, 1.4) in DNPT (*p* = 0.03) compared to standard care group. After 48 hours, eight patients (9%) in standard care were on step 4 of the modified analgesia ladder, compared to nine (8%) with a device (*p* > 0.99) of which three patients (6%) were in the DN group and six patients (11%) were in the DNPT group. Table 6 summarises patient-reported outcomes using the IPOQ at 48 hours. The opioid prescription on discharge in SC vs. DN and DNPT groups is described in Table 9.

## 4. Discussion

### 4.1. Role of a Device on Pain Outcomes

This study confirms that poorly controlled postoperative pain remains a problem, with 45% of patients in standard care reporting moderate or severe pain on the day following surgery, consistent with previously published data [1, 2, 37]. We investigated whether a multifunctional device, allowing free reporting of pain with content tailored to patients' operative pathways, could improve pain and patient experience and reduce discharge opioid use and length of stay after surgery. Our primary objective (1) was to assess the efficacy of using a device on poor pain control at 24 and 48 hours. Patients with a device were over two times less likely to report poor pain control after 24 hours than those in standard care. At 48 hours, incidence of poor pain control improved in both standard care and device groups, with no significant difference between them.

We aimed to determine the efficacy of the device on proportion of time spent in severe pain and satisfaction with pain treatment (2A). After 48 hours, patients with a device reported 10% less time spent in severe pain than did those with standard care. As seen in multiple previous studies, many patients had moderate or severe pain 24 hours after surgery [37], yet they recorded high satisfaction with care [38]; whilst all groups reported high satisfaction, device groups rated satisfaction significantly higher than those in standard care group. Furthermore, IPOQ individual ratings of anxiety, helplessness, patient involvement in decision making, and patient information were improved after 48 hours with a device, although they were generally not in a problematic range for any group. A further objective was to assess the effect of a device on opioid prescription at discharge and length of stay (2B). Patients without a device were twice as likely to be prescribed strong opioids on discharge and half as likely to be prescribed weak opioids. There was no difference in length of in-hospital stay between device and standard care groups. We aimed to assess patient satisfaction and experience with the device (2C). The device was overall rated well by patients, and informal comments indicated that they used the content to help manage emotional as well as more sensory aspects of pain for which analgesics are the usual resort, for instance, returning to information about pain and the surgery for reassurance. Finally, we sought to determine the effect of feedback to nursing staff and/or the pain team on the patient's pain experience, discharge opioid prescription, and length of stay (2D). Only patients with feedback to both the pain team and nursing staff showed improvements in pain control at 24 hours, whereas those patients with feedback to nursing staff only during these changes did not differ from those receiving standard care. Both groups with a device showed less time in severe pain and higher satisfaction than those in standard care. Neither device group showed improved analgesia over standard care at 48 hours.

Mobile applications may improve pain, distress, and quality of life in varying chronic pain states [27, 29, 39–41] but to date have limited evidence for use in the perioperative setting. In this study, we have shown that a device with a bespoke application tailored to patients' perioperative journeys can reduce pain at 24 hours; at 48 hours, fewer patients across all groups reported poorly controlled pain, and there was no difference between groups.

In this study, access to a multifunctional digital device with self-management options for pain control was associated with reduced prescription of strong opioids on discharge from hospital, with improved pain control and patient satisfaction. Several perioperative interventions, including patient and clinician education and computerised decision support tools, have been shown to reduce opioid prescription at discharge whilst maintaining good analgesia [42]. Severe pain in the perioperative period and prescription of strong opioids at discharge are risk factors for long term opioid use, so encouraging the use of nonpharmacological methods for pain control from the start could reduce risk [6, 43, 44]. A large proportion of our patients accessed nonpharmacological means of acute pain management, especially material on pain and surgical information and guided relaxation exercises. Further studies are required to test hypotheses concerning the psychological mechanisms by which better pain control was achieved and to record use of specific functions of the device in relation to this.

### 4.2. Role of Feedback on Pain Control

Applications with clinician notification can improve outcomes for patients with chronic pain [28], but similar results have emerged in hospitalised patients. We had hypothesised that having access to a device to self-report pain would lead to improved analgesia by improving staff responsiveness, with a more marked effect when a specialist pain team was alerted [45]. The results provided partial support for this, but other psychological mechanisms, such as greater perceived control over pain by using the device, might have fostered improved pain scores [46]. By adjusting for baseline differences, the DNPT group (with feedback to nursing staff and pain team), but not the DN group (with feedback to nursing staff alone), had better pain control at 24 h, but not at 48 h, when pain control for all groups had improved over that at 24 h. Both device groups had a lower percentage of time in severe pain at 48 h than did the standard care group, and at discharge, strong opioid use was lower in DNPT than in standard care group. Thus, real time feedback of patients' pain scores to nursing staff and a pain team appears to have improved postoperative pain control.

The extra value of feedback to the pain team is demonstrated by patients in the DNPT group having almost three times lower odds of poor pain control at 24 hours than those in standard care. The absence of difference in the proportion of patients with poor pain control between the DN and SC groups suggests that staff who were nursing both DN and SC groups had already exhausted their resources for reducing pain, and referral to the pain team was necessary to improve pain control further.

Furthermore, having a device reduced the amount of time patients spent in severe pain (by up to 42%) and improved overall satisfaction with pain control. Importantly, patients with a device that fed back to both nursing staff and pain team were substantially less likely to be prescribed strong opioids on discharge than those without a device. Those patients with feedback to nursing staff alone had a nonsignificant reduction in strong opioids at discharge with an increase in prescription of any opioid analgesic, suggesting substitution of strong by weak opioids. These findings are accompanied by a small but statistically significant advantage in patient satisfaction in both groups with the device at 48 hours. Whilst we are unable to make strong conclusions regarding the mechanism through which the device enabled improved pain control and reduced opioid prescription at discharge, in addition to the nonpharmacological methods available on the device for pain management, we hypothesise that increased alerting of healthcare professionals including the pain team to poor pain control allows for earlier intervention and appropriate escalation of analgesia. This in turn reduces likelihood of poorly controlled pain and time spent in severe pain. Furthermore, this allows earlier deescalation of strong opioids when appropriate, helping prevent inappropriate prescription of strong opioids on discharge. This may in part explain the significant improvements in pain at 24 hours, time in severe pain at 48 hours, and frequency of strong opioid prescription at discharge amongst the group with device feedback to the pain team as well as the nursing staff, rather than nursing staff alone.

### 4.3. Limitations

Due to the difficulties of maintaining blindness of staff assessing and treating pain to treatment allocation, this study was not randomised. This means that biases may have been introduced by different behaviour of staff towards patients with and without devices, from recruitment to differential efforts to reduce pain, although no ward staff were directly involved in this study. Patients were allocated to groups in series, according to time of recruitment. This may introduce bias due to changing hospital practice and healthcare professional prescribing over time. Although it was possible that standard care improved over the time in which the three cohorts were recruited, there was no specific hospital initiative with these aims, and the extent of poor control found in our study at baseline, 24 hours after surgery, was similar in standard care and device groups, suggesting that if there were such a change, it would not be a large one. Additionally, the healthcare professionals responsible stayed consistent over the period of the trial, meaning it was unlikely without an education programme that prescribing practice would change greatly over time. Furthermore, we controlled for incidental baseline differences between groups.

There may also have been premorbid medical and psychosocial differences not assessed that could have contributed to the likelihood of developing poorly controlled postoperative pain [47], but neither pain, preoperative medication use, anxiety, or depression scores differentiated groups at baseline. A previous study showed that using a similar device reduced anxiety [20], mitigating the pain experience [48], but no such differences were found in those populations being older, undergoing more invasive surgery, and experiencing more severe postoperative pain. Whilst verbal and numerical rating scales have good validity in older patients [49, 50], those patients tend to underreport pain and possibly pain-related anxiety [51, 52]; in this study, assessment of pain was on a simple scale, brief and unidimensional, but one that is easy to request verbally, making data more complete. Reporting pain on a device could avoid biases introduced by the clinician-patient relationship [53], but an older population may be less inclined to use digital technology than a younger group [54–56], as may those whose surgery has a greater impact [57]. These factors may have influenced the outcomes of this study, and a more fully controlled, randomised comparison of use of the device with standard care is desirable.

Furthermore, since feedback to the pain team occurred in this study only between 8 am and 5 pm, larger differences between device groups might be evident if pain team expertise had been available at all hours. We cannot rule out the possibility that the lack of specialised response to reports of severe pain out of hours may have discouraged some patients from reporting their pain using the device.

### 4.4. Future Directions

Whilst this study was carried out at a specialist thoracic and urology surgical centre, severe postoperative pain is found across surgical specialities [58, 59]. The rate of poorly controlled postoperative pain in this cohort of patients is comparable to that seen in previous studies [59]. Furthermore, many of these other surgical procedures are associated with the risk of chronic opioid therapy [60, 61]. Integrating this service into surgical specialities would allow immediate feedback of poor pain control to healthcare professionals around the clock, and ensuring a response to poor pain control via the device at any time may further empower patients, leading to improved trust. A device such as this may also foster personalised care, identifying those patients who require further input from specialist pain teams and limiting routine reliance on opioids.

Technology is commonly promoted as the solution for overstretched healthcare systems, but it requires support from clinical and nonclinical services for reliable implementation and effectiveness. Availability of these functions as apps on patients' own devices could improve management of pain persisting after surgery by promoting nonpharmacological methods of pain management before and immediately after surgery as well as after discharge. Thorough assessment of the impact of the device on these factors is required before integration into clinical practice.

## Figures and Tables

**Table 1:** Modified analgesic ladder.

Step	Analgesia
Step 1	Simple analgesia
Step 2	Step 1 + weak opioid
Step 3	Step 1 or 2 + strong opioid
Step 4	PCA/regional anaesthesia infusion technique

**Table 2:** Study measures summary

Measure	Outcome
Pain score (worst pain in preceding 24 hours, 0–4) at 24 and 48 hours	Primary
Patient-reported time in severe pain and satisfaction with pain treatment (measured in IPOQ) at 48 hours	Secondary
International Pain Outcome Questionnaire (IPOQ) at 48 hours	Secondary
Analgesia administered (using modified analgesic ladder) at 48 hours	Secondary
Medication use on discharge and length of stay	Secondary
Device evaluation questionnaire	Secondary

IPOQ: International Pain Outcome Questionnaire.

**Table 3:** Content accessible via MServ home screen.

“Home screen” icons	Content
Submit Pain Score	Quick link for patient to submit pain score
Pain Information	Patient information videos and leaflets on advice from pain team; pain mechanisms; pain psychology; pain management as inpatient and at home
Self Help	Patient information videos on guided physiotherapy; mindfulness; relaxation
My Operation	Patient information videos tailored to patient's perioperative journey, e.g., “patient information on chest drains” or “going home with a catheter” depending on surgery type
Entertainment	Audiobooks; games; puzzles

**Table 4:** Summary statistics are number (percentage), median [interquartile range], or mean ± standard deviation.

	Category	SC (*n* = 102)	Device (*n* = 132)	DN (*n* = 66)	DNPT (*n* = 66)	SC vs. device *p* value
Sex	Female	44 (43%)	58 (44%)	31 (47%)	27 (41%)	0.90
	Male	58 (57%)	74 (56%)	35 (53%)	39 (59%)	
Age	n/a	60.3 ± 15.7	61.2 ± 15.0	63.5 ± 15.3	58.9 ± 14.6	0.64
Speciality	Thoracic	77 (75%)	124 (94%)	63 (95%)	61 (92%)	**<0.001**
	Urology	25 (25%)	8 (6%)	3 (5%)	5 (8%)	
Preadmission opioid use	Any	20 (20%)	20 (15%)	9 (14%)	11 (17%)	0.37
	Strong	8 (8%)	6 (5%)	3 (5%)	3 (5%)	0.29
Surgery type: major or complex	n/a	99 (97%)	126 (95%)	61 (92%)	65 (98%)	0.53
Baseline pain score ≥ 2/4	n/a	88 (87%)	122 (93%)	58 (89%)	64 (99%)	0.12
% reported time in severe pain	n/a	30 [10, 70]	10 [0, 30]	10 [0, 40]	10 [10, 20]	**<0.001**
Post-op step 4 analgesia	No	26 (25%)	5 (4%)	3 (5%)	2 (3%)	**<0.001**
	Yes	76 (75%)	124 (96%)	63 (95%)	64 (97%)	

**Table 5:** Pain control at 24 and 48 hours in standard care group and device groups.

Time point	SC	Device	Unadjusted: SC vs. device		Adjusted: SC vs. device	
	*n*/*N* (%)	*n*/*N* (%)	OR (95% CI)	*p*-value	OR (95% CI)	*p* value
24 hours	46/101 (45%)	35/125 (28%)	0.46 (0.27, 0.81)	**0.007**	0.45 (0.25, 0.81)	**0.004**
48 hours	22/90 (24%)	20/110 (18%)	1.46 (0.74, 2.89)	0.28	0.72 (0.34, 1.50)	0.38

Number (percentage) of patients with poor pain control (score >1/4) in standard care (SC) group and both device groups, and odds ratio between standard care group and both device groups, unadjusted and adjusted for age, speciality, and baseline PCA use.

**Table 6:** International Pain Outcome Questionnaire (IPOQ).

Patient-reported outcome	Scale	SC	Device	DN	DNPT	*p* value
		(*n* = 90)	(*n* = 108)	(*n* = 52)	(*n* = 56)	
Worst pain	(0–10)	6 [3.25, 8.75]	7 [5, 8]	7 [5, 8]	6.5 [5, 8]	0.82
Least pain	(0–10)	1 [0, 3]	2 [1, 3]	2 [1, 3]	2 [1, 3]	0.06
Percent time in severe pain	(0–100%)	10 [0, 38]	0 [0, 10]	0 [0, 10]	0 [0, 10]	<0.001
Interference: in bed	(0–10)	4 [1.25, 7]	5 [3, 7]	6 [3, 7]	5 [3, 7]	0.29
Interference: breathing	(0–10)	4.5 [1, 8]	5 [3, 7]	5 [3, 7]	5 [4, 7]	0.39
Interference: sleeping	(0–10)	0.5 [0, 6]	0 [0, 3]	0 [0, 2]	1 [0, 4]	0.26
Has been out of bed?	“Yes”	86/90 (96%)	6/104 (95%)	49/54 (91%)	55/56 (98%)	0.74
Interference: out of bed activities	(0–10)	2 [0, 5]	1 [0, 3]	1 [0, 3]	1 [0.5, 3]	0.65
Anxiety	(0–10)	0 [0, 4]	0 [0, 0]	0 [0, 1]	0 [0, 0]	<0.001
Helplessness	(0–10)	0 [0, 5]	0 [0, 0]	0 [0, 0]	0 [0, 0]	<0.001
Nausea	(0–10)	0 [0, 2]	0 [0, 4]	0 [0, 2]	0 [0, 4]	0.18
Drowsiness	(0–10)	0 [0, 4]	0 [0, 4]	0 [0, 3]	0 [0, 5.25]	0.83
Itching	(0–10)	0 [0, 2]	0 [0, 0]	0 [0, 0]	0 [0, 0]	0.03
Dizziness	(0–10)	0 [0, 1]	0 [0, 0]	0 [0, 0]	0 [0, 0]	0.47
Pain relief	(0–100%)	90 [70, 100]	90 [80, 90]	90 [80, 90]	90 [80, 90]	0.94
Would like more treatment?	“Yes”	20/90 (22%)	21/110 (19%)	10/54 (19%)	11/56 (20%)	0.72
Would like more information regarding treatment options?	“No”	65/90 (72%)	108/110 (98%)	54/54 (100%)	54/56 (96%)	<0.001
Involvement in decision making	(0–10)	8 [2, 10]	10 [9, 10]	10 [8, 10]	10 [7.75, 10]	<0.001
Satisfaction with treatment	(0–10)	10 [1, 10]	10 [8, 10]	10 [1, 10]	10 [1, 10]	0.31

Measures of pain severity, interference, mood, side effects, and involvement and satisfaction with treatment in the previous 24 hours after 48 hours of trial (exit) in standard care (SC) and device groups (pooled, DN, and DNPT). Data are displayed as number (percentage) or median [interquartile range] as appropriate.

**Table 7:** Opioid prescription on discharge in standard care (SC) group and both device groups.

Opioid	SC	Device	Device vs. SC
	*n* (%)	*n* (%)	OR (95% CI)
All opioids	80 (78%)	115 (88%)	2.04 (1.00, 4.14)
			*p* = **0.05**
Strong opioid	23 (22%)	13 (10%)	0.39 (0.18, 0.84)
			*p* = **0.02**

Number of patients (percentage) prescribed opioids in SC and device groups and odds ratio (95% CI) of opioid prescription on discharge in device groups compared to standard care SC. Odds ratios were adjusted for admission opioid use.

**Table 8:** Poor pain control at 24 and 48 hours in standard care group and groups with feedback to healthcare professionals.

Time point	SC	DN	DNPT	DN vs. SC		DNPT vs. SC	
	*n*/*N* (%)	*n*/*N* (%)	*n*/*N* (%)	OR (95% CI)	*p* value	OR (95% CI)	*p* value
24 hours	46/101 (45%)	19/61 (31%)	16/64 (25%)	0.53 (0.26, 1.08)	0.08	0.33 (0.16, 0.70)	**0.004**
48 hours	22/90 (24%)	9/54 (17%)	11/56 (20%)	0.63 (0.27, 1.60)	0.33	0.65 (0.27, 1.57)	0.34

Odds ratios for number (%) of patients with poor pain control (pain score >1/4) for DN (device with feedback to nursing staff) and DNPT (device with feedback to nursing staff and pain team) groups compared with standard care group (SC), at 24 and 48 hours.

**Table 9:** Opioid prescription on discharge in standard care (SC), DN, and DNPT groups.

Opioid	SC	DN	DNPT	DN vs. SC	DNPT vs. SC
	*n* (%)	*n* (%)	*n* (%)	OR (95% CI)	OR (95% CI)
All opioids	80 (78%)	60 (92%)	55 (83%)	3.44 (1.23, 9.66)	1.40 (0.63, 3.14)
				*p* = **0.02**	*p* = 0.41
Strong opioid	23 (22%)	7 (11%)	6 (9%)	0.43 (0.17, 1.11)	0.35 (0.13, 0.95)
				*p* = 0.08	*p* = **0.04**

Number of patients (percentage) prescribed opioids in SC, DN, and DNPT groups and odds ratio (95% CI) of opioid prescription on discharge in device groups compared to standard care (SC). Odds ratios were adjusted for admission opioid use. Opioids were defined according to Supplementary Table 1.

## Data Availability

The data used to support the findings of this study are available on request from the corresponding author.

## Supplementary Materials

Supplementary Materials Supplementary Table 1: definition of strong and weak opioids. Supplementary Table 2: prescription of nonopioid medication used at home prior to admission. Number (percent) of patients prescribed in standard care (SC) and device groups (DN and DNPT). Supplementary Table 3: prescription of nonopioid medication on discharge from hospital. Number (percent) of patients prescribed in standard care (SC) and device groups (DN and DNPT).
