# Supplementary material for: An Interactive Pain Application (MServ) Improves Postoperative Pain Management
Source: Pain Res Manag. 2021 Apr 2;2021:8898170. doi: 10.1155/2021/8898170 (PMC8035036; doi:10.1155/2021/8898170)
Supplement: Supplementary Materials — Supplementary Table 1: definition of strong and weak opioids. Supplementary Table 2: prescription of nonopioid medication used at home prior to admission. Number (percent) of patients prescribed in standard care (SC) and device groups (DN and DNPT). Supplementary Table 3: prescription of nonopioid medication on discharge from hospital. Number (percent) of patients prescribed in standard care (SC) and device groups (DN and DNPT). [file 8898170.f1.docx]

# Supplementary Information

| **Weak Opioids (Any Dose)** | **Strong Opioids (Any Dose)** |
| --- | --- |
| Codeine (Oral) | Morphine (Immediate Release, Oral) |
| Dihydrocodeine (Oral) | Morphine (Modified Release, Oral) |
| Co-Codamol (Oral) | Oxycodone (Immediate Release, Oral) |
| Co-Dydramol (Oral) | Oxycodone (Modified Release, Oral) |
| Tramadol (Oral) | Fentanyl (Transdermal) |
|  | Buprenorphine (Transdermal) |
| Any opioids, preparations or routes of administration not listed were not prescribed at admission nor discharge. | |

**Supplementary table 1. Definition of strong & weak opioids**

|  | **Medications pre-admission** | | |
| --- | --- | --- | --- |
|  | **SC** | **DN** | **DNPT** |
| **Simple Analgesics** | 18 (18%) | 8 (12%) | 19 (29%) |
| **Anti-Neuropathics** | 9 (9%) | 5 (8%) | 6 (9%) |
| **Antidepressants** | 9 (9%) | 11 (16%) | 11 (16%) |
| **Benzodiazpines** | 3 (3%) | 5 (8%) | 5 (8%) |

**Supplementary table 2. Prescription of non-opioid medication used at home prior to admission**. Number (Percent) of patients prescribed in standard care (SC) and device groups (DN & DNPT).

|  | **Medications on discharge** | | |
| --- | --- | --- | --- |
|  | **SC** | **DN** | **DNPT** |
| **Simple Analgesics** | 90 (90%) | 61 (92%) | 62 (92%) |
| **Anti-Neuropathics** | 7 (7%) | 5 (7%) | 6 (9%) |
| **Antidepressants** | 6 (6%) | 10 (15%) | 10 (15%) |
| **Benzodiazpines** | 2 (2%) | 5 (7%) | 3 (5%) |

**Supplementary table 3. Prescription of non-opioid medication on discharge from hospital.** Number (Percent) of patients prescribed in standard care (SC) and device groups (DN & DNPT).
